# Bioengineered bacterial outer membrane vesicles encapsulated Polybia–mastoparan I fusion peptide as a promising nanoplatform for bladder cancer immune-modulatory chemotherapy

**DOI:** 10.3389/fimmu.2023.1129771

**Published:** 2023-03-14

**Authors:** Chunyu Ren, Yangyang Li, Zhaoqing Cong, Zhuoran Li, Leiming Xie, Song Wu

**Affiliations:** ^1^ Medical College, Anhui University of Science and Technology, Huainan, Anhui, China; ^2^ Institute of Urology, The Third Affiliated Hospital of Shenzhen University, Shenzhen, Guangdong, China; ^3^ Shenzhen Following Precision Medical Research Institute, Luohu Hospital Group, Shenzhen, Guangdong, China; ^4^ South China Hospital, Health Science Center, Shenzhen University, Shenzhen, Guangdong, China

**Keywords:** outer membrane vesicles, polybia-mastoparan I, antitumor platform, bladder cancer, anti-tumor immune response

## Abstract

**Background:**

Nanosized bacterial outer membrane vesicles (OMVs) secreted by Gram-negative bacteria have emerged as a novel antitumor nanomedicine reagent due to their immunostimulatory properties. The encapsulated bacterial composition in OMVs can be edited *via* manipulating bioengineering technology on paternal bacteria, allowing us to design an ingenious antitumor platform by loading the Polybia–mastoparan I (MPI) fusion peptide into OMVs.

**Methods:**

OMVs containing the MPI fusion peptide were obtained from bioengineered *Escherichia coli* transformed with recombinant plasmid. The antitumor efficacy of bioengineered OMVs *in vitro* was verified by performing cell viability and wound-healing and apoptosis assays using MB49 and UMUC3 cells, respectively. Subcutaneous MB49 tumor-bearing mice were involved to investigate the tumor inhibition ability of bioengineered OMVs. Moreover, the activated immune response in tumor and the biosafety were also evaluated in detail.

**Results:**

The resulting OMVs had the successful encapsulation of MPI fusion peptides and were subjected to physical characterization for morphology, size, and zeta potential. Cell viabilities of bladder cancer cells including MB49 and UMUC3 rather than a non-carcinomatous cell line (bEnd.3) were decreased when incubated with bioengineered OMVs. In addition, bioengineered OMVs restrained migration and induced apoptosis of bladder cancer cells. With intratumor injection of bioengineered OMVs, growths of subcutaneous MB49 tumors were significantly restricted. The inherent immunostimulation of OMVs was demonstrated to trigger maturation of dendritic cells (DCs), recruitment of macrophages, and infiltration of cytotoxic T lymphocytes (CTLs), resulting in the increased secretion of pro-inflammatory cytokines (IL-6, TNF-α, and IFN-γ). Meanwhile, several lines of evidence also indicated that bioengineered OMVs had satisfactory biosafety.

**Conclusion:**

Bioengineered OMVs fabricated in the present study were characterized by strong bladder cancer suppression and great biocompatibility, providing a new avenue for clinical bladder cancer therapy.

## 1 Introduction

Bladder cancer (BCa) has become the most prevalent urological malignancy worldwide, in which approximately 75% are initially diagnosed as non-muscle-invasive BCa in the clinical setting (1, 2). Although treated with the current standard therapeutic regimen (transurethral surgical intervention followed by regular intravesical instillation therapy) (3–5), nearly 75% of patients still suffer from cancer recurrence or malignant progression within 5 years, leading to lifelong care and high treatment costs (6, 7). The rather poor prognosis warrants additional urgent treatment strategies to improve the current dilemma of BCa treatment.

An antibiotic peptide named Polybia–mastoparan I (MPI), derived from the venom of the social wasp *Polybia paulista*, is composed of 14 amino acids (IDWKKLLDAAKQIL) with an α-helix conformation (8, 9). In contrast to normal mammalian cell membranes consisting of zwitterionic phospholipids usually with neutral charge, cancer cell membranes carry a net negative charge due to the existence of abundant anionic phospholipids such as phosphatidylserine (three- to sevenfold more than normal mammalian cell membranes) (10–12). Due to electrostatic interactions and hydrophobic effect, the cationic anticancer peptide MPI could easily insert into the bilayer of cell membrane and enable membranes lysis, causing irreversible damage to cancer cells (13, 14). Recently, MPI has been demonstrated to exhibit preferential toxicity to several BCa cell lines while having little damage to nontumorigenic cells (9, 15, 16). In our previous report, chemosynthetic MPI serving as chemotherapy agents has been encapsulated into transmucosal nanoparticles for intravesical instillation therapy of BCa, achieving satisfactory therapeutic effects (16). Therefore, it is of great significance to develop MPI peptides as superior chemotherapy agents to ameliorate BCa treatment outcomes.

Gram-negative bacteria can naturally germinate nanoscale vesicles from their outer membranes, which have been well defined as outer membrane vesicles (OMVs), and secrete into surroundings to accomplish various physiology and pathogenesis courses such as horizontal gene transfer, toxin elimination, and immune adjustment (17, 18). The versatile OMVs have received increasing attention in recent years on account of their recognized advantages in vaccine development and therapeutic drug delivery (18, 19). Bacteria-derived OMVs have been confirmed as an emerging immune adjuvant benefiting from their abundant pathogen-associated molecular patterns (PAMPs), which enable the activation of anticancer immune response (20–22). Compared to attenuated bacteria vaccines, the acellular OMVs represent better effectiveness and safety when applied in the interior of an organism (21). Moreover, the biological membranes derived from OMVs have also been utilized as immunostimulatory biomaterials to coat nanoparticles for drug delivery (23–25). Genetic modification at the C terminal of the outer membrane protein cytolysin A (ClyA) allows OMVs to display multiple functional components on their surface, achieving specific vaccine platform for cancer therapy or enhanced tumor targeting (26–29). Regarding the nano-carrier exploration, exogenous genetic substances such as plasmids (29) and siRNAs (28, 30) can be facilely loaded into OMVs by the electroporation technique due to the phospholipid bilayer construction, and ultimately delivered for gene therapy. Manipulating genetically engineered bacteria to produce OMV-encapsulated therapeutic protein agents has been confirmed with cogent feasibility (31–35). Therefore, the development of bioengineered OMVs containing an anticancer peptide provides a novel area to be explored for cancer treatment.

In this work, we report a bioengineered OMV-based platform facilely fabricated by wrapping genetically generated fusion MPI peptides into OMVs for BCa immune-modulatory chemotherapy, resulting in satisfactory therapeutic outcome and biosafety. Through transforming a recombinant plasmid designed to express MPI at the N or C terminal of the fusion peptide, we obtained different bioengineered OMVs from transformed *Escherichia coli* cells. The enhanced BCa inhibition capacity of these bioengineered OMVs was verified by a series of *in vitro* and *in vivo* experiments. Furthermore, we also demonstrated the activated immune response in tumor microenvironment induced by OMVs. Biocompatibility assessment indicated superb biosafety of the administered OMVs. Our research proposes a significant strategy to expanding applications of MPI peptide and OMVs in cancer treatment.

## 2 Materials and methods

### 2.1 Construction of plasmids

To construct the plasmid that can generate fusion protein with MPI at the N terminal (MPI-N), the codon-optimized DNA sequence encoding MPI (MPI-N-sense and MPI-N-antisense shown in **Table 1**) was synthesized by IGE Biotech Co., Ltd (Guangzhou, China) and then cloned into a commercial plasmid vector pET43.1a (Novagen, Merck KGaA) located between *Nde* I and *Spe* I endonuclease sites. As for the plasmid expressing fusion protein with MPI at the C terminal (MPI-C), the gene encoding eGFP was amplified from a saved plasmid in our laboratory and inserted into pET43.1a using *Nde* I and *BamH* I endonuclease. Subsequently, the synthesized DNA sequence of MPI including a terminator TAA (MPI-C-sense and MPI-C-antisense shown in **Table 1**) was attached behind eGFP gene using *BamH* I and *Xho* I endonuclease. In order to add the signal peptide (SP) of OmpA (MKKTAIAIAVALAGFATVAQA, searching from https://www.uniprot.org/) at the N terminal of MPI-N, PCR was adopted two times to fuse the DNA sequence of SP and MPI with pET43.1a-MPI-N as the template. Primers of SP-Forward-2 and SP-Reverse shown in **Table 1** were used in the first PCR, and SP-Forward-1 and SP-Reverse were added for the second PCR. All the recombinant plasmids were confirmed by Sanger sequencing (IGE Biotech, Guangzhou, China) before transformation.

**Table 1:** Primers used to construct MPI-N, MPI-C, and MPI-SP expression plasmids.

Primer name	Sequence (5’-3’)
MPI-N-sense	TATG**ATCGACTGGAAAAAACTGCTGGACGCTGCGAAACAAATCCTG**GGTGGTGGTG
MPI-N-antisense	CTAGTACCACCACC**CAGGATTTGTTTCGCAGCGTCCAGCAGTTTTTTCCAGTCGAT**TA
MPI-C-sense	GATCCATCGAAGGTCGT**ATTGACTGGAAAAAACTGCTGGATGCTGCTAAGCAGATTCTC**TAAC
MPI-C-antisense	TCGAGTTA**GAGAATCTGCTTAGCAGCATCCAGCAGTTTTTTCCAGTCAAT**ACGACCTTCGATG
SP-Forward-1	GGGTTTCATATGAAAAAGACAGCTATCGCGATTGCAGTGGCACTGGCTGGTTTCGCTAC
SP-Forward-2	GGCTGGTTTCGCTACCGTAGCGCAGGCCGGTGGTGGTACTAGTATCGACTGGAAAAAAC
SP-Reverse	CCGCTCGAGCTGCAGGTAC

DNA sequences with bold indicate MPI encoding sequences. DNA sequences with underline indicate pairing sequence for the second PCR.

### 2.2 OMV preparation

After being transformed with recombinant plasmid (pET43.1a-MPI-N, pET43.1a-MPI-C, or pET43.1a-MPI-SP), the *E. coli* (BL21) strain was screened and maintained on the solid Luria Broth (LB) plate with ampicillin (100 μg/ml). To induce the expression of fusion protein, a single transformed bacterial colony on LB plate was transferred into liquid LB medium supplemented with 100 μg/ml of ampicillin and cultured at 37°C with shaking at 220 rpm. When the bacterial growth reached the mid-log phase (OD_600_: 0.6–0.8), 0.5 mM of isopropyl β-D-1-thiogalactopyranoside (IPTG) was added to the medium and continuously cultured at 18°C with shaking at 180 rpm for a further 12 h or overnight.

OMV pellets were prepared from the transformed *E. coli* after the expression of fusion protein was induced by IPTG according to the description in a previous report with some modifications (36). Briefly, the bacteria were collected by centrifugation at 5,000×*g* for 10 min and resuspended in sterile PBS solution (pH 7.4) with the concentration at 1 g (wet weight) of bacteria in 5 ml of PBS. The bacteria suspension was subjected to a sonication treatment under a power of 65 W for 20 min with repetitive 3-s pulses and a 5-s interval by an ultrasonic Homogenizer (SCIENTZ-IID, Ningbo, China). During this process, an ice bath was necessary to maintain the cold temperature of the bacterial suspension. Afterwards, bacteria were removed by two centrifugations at 10,000×*g* at 4°C for 20 min, and the supernatant was filtered through 0.2-μm polyvinylidene fluoride filters (Jet Biofil, Guangzhou, China) to mitigate intact bacteria or cell debris. Lastly, OMVs were isolated by ultracentrifugation at 150,000×*g* for 2 h at 4°C (Optima XPN-100, Beckman Coulter). The purified OMVs were resuspended in moderate PBS solution and stored at −20°C until use. The bicinchoninic acid (BCA) protein assay kit (Beyotime, Shanghai, China) was applied to quantify the total protein concentration of OMVs as per the manufacturer’s instruction.

### 2.3 OMV characterization

After the OMV samples were fixed by 3% glutaraldehyde and negatively stained with 2% uranyl acetate, transmission electron microscopy (TEM) analysis was performed on a JEM-1200 EX instrument with an accelerating voltage of 120 kV to obtain the morphology characterization of OMVs. The particle size distributions and zeta potentials of OMVs were measured by Zetasizer Nano ZS (Zetasizer Nano ZS-90, Malvern, UK).

### 2.4 Fusion peptide analysis

The protein samples of bacteria and OMVs were prepared using radio immunoprecipitation assay (RIPA) lysis buffer containing 1% protease inhibitor. For bacteria that was difficult to lyse, a further sonication treatment was employed. After the protein concentrations were quantified with the BCA protein assay kit (Beyotime, Shanghai, China), the protein components were separated by electrophoresis in a 12% polyacrylamide gel. The polyacrylamide gel was incubated in Coomassie blue R250 solution at room temperature for 1 h and then incubated in the destaining solution until there are clear bands for imaging to identify MPI-C, MPI-N, and MPI-SP.

To detect MPI-N and MPI-SP by Western blot analysis, the protein components in polyacrylamide gel were transferred to a polyvinylidene difluoride (PVDF) membrane (Millipore). The transferred membrane was incubated with anti-His antibody (1:2,000, Beijing Ray Antibody Biotech) at room temperature for 2 h or at 4°C overnight after being blocked with 5% skim milk. Subsequently, the membrane was incubated with horseradish peroxidase (HRP)-conjugated secondary antibody at room temperature for 1 h after three washes with the TBST buffer. Before chemiluminescence detection by a luminescence imaging system (GelView6000 Pro, Guangzhou Biolight Biotechnology Co., Ltd., China), three washes with the TBST buffer were again performed to remove dissociative secondary antibody.

### 2.5 Cell lines

Murine BCa cell line MB49, human BCa cell line UMUC3, and mouse brain microvascular endothelial cell line bEnd.3 were purchased from the American Type Culture Collection (ATCC, Shanghai). Cells were routinely cultured in a humidified incubator (Thermo Fisher Scientific) at 37°C with 5% CO_2_. MB49 cells were grown in Roswell Park Memorial Institute (RPMI) 1640 medium containing 10% fetal bovine serum (Gibco Life Technologies) and UMUC3 and bEnd.3 cells were cultured in Dulbecco’s Modified Eagle Medium (DMEM) supplemented with 10% fetal bovine serum (Gibco Life Technologies). In addition, 100 U/ml of penicillin and 100 mg/ml of streptomycin were also added into the culture medium before use.

### 2.6 OMVs uptake by MB49 cells

When the confluence density of MB49 cells grown in six-well plates reached approximately 80%, the medium was replaced by a fresh medium containing 100 μg/ml of DiL-labeled OMV^MPI-C^, in which eGFP was expressed. After another 2-hour incubation, the OMV^MPI-C^ phagocytized by MB49 cells were observed under a confocal laser scanning microscope (CLSM) with a 40× oil objective lens (Zeiss LSM800 with Airyscan, Germany).

### 2.7 Cytotoxicity assays

Cytotoxicity assays were performed by using Cell Counting Kit-8 (Meilunbio, Dalian, China) following the manufacturer’s protocols. MB49, UMUC3, or bEnd.3 cells were seeded in a 96-well plate with 1 × 10^4^ cells/well. When cells attached to the bottom of plate, the medium was replaced with the fresh medium containing 10, 30, 50, 100, 200, or 300 μg/ml of OMV^WT^, OMV^MPI-N^, OMV^MPI-SP^, or OMV^MPI-C^. The equivalent volume of PBS was added as a negative control group. The cell viabilities were detected after 24-h incubation with a Spark^®^ Multimode Microplate Reader (TECAN, Switzerland) by measuring the absorbance at 450 nm.

### 2.8 Wound-healing assay

With regard to the wound-healing assay, tumor cells were plated into a 24-well plate (1 × 10^5^ cells/well). When the cell monolayers were wounded by a gap, the same OMV treatment as described above was performed. The wound-healing degrees were observed under an inverted microscope (Zeiss Axio Observe, Germany) at different time points (0, 12, and 24 h). Wound closures were calculated by the following formula:

### 2.9 Apoptotic tumor cell detection

For apoptosis detection of tumor cells, MB49 and UMUC3 cells cultured in six-well plates were treated with different formulations of OMVs as described above. The tumor cells were collected after 48 h and stained using Annexin V-Alexa Fluor 488/PI Apoptosis Detection Kit (Beyotime, Shanghai, China) as per the manufacturer’s instructions. The apoptosis cells were analyzed on a flow cytometry (FCM) instrument (BD FACS Calibur).

### 2.10 Antitumor investigation of bioengineered OMVs *in vivo*

All animal experiments in this study were performed under the regulatory supervision of Animal Use and Care Administrative Advisory Committee of Shenzhen Luohu Hospital Group. C57BL/6 mice (male, 6–8 weeks) were recruited to establish a subcutaneous BCa model to evaluate the anticancer efficacy of the bioengineered OMVs. Specifically, MB49 cells (1 × 10^6^) were subcutaneously inoculated into the right flank of C57BL/6 mice and the generated tumors were allowed to reach ~100 mm^3^. Then, tumor-bearing mice were randomly divided into five experimental groups with five mice in each group and treated with different formulations: (1) PBS, (2) OMV^WT^, (3) OMV^MPI-N^, (4) OMV^MPI-SP^, and (5) OMV^MPI-C^. Treatment was repeated every 3 days for a total of four times. For OMV administration, each mouse was intratumorally injected with 50 μl of solution containing 100 μg of vesicle protein. Tumor volumes were routinely measured by a vernier caliper every other day and calculated as 0.5 × LW^2^, where L and W represent the longest and shortest axes of the tumor, respectively. In the meantime, body weights of tumor-bearing mice were also recorded during the treatment course.

At the end of treatment on day 15, all mice were euthanized after blood samples were collected by orbit for blood routine examination. Moreover, the serum levels of aspartate transaminase (AST), alanine aminotransferase (ALT), alkaline phosphatase (ALP), and blood urea nitrogen (BUN) were detected with a Biochemical Autoanalyzer (Chemray 800, Rayto, Shenzhen, China). Tumor tissues and major organs including heart, liver, spleen, lung, and kidney were excised for further analyses. Tumors were immediately recorded by imaging and weighing after surgical resection. For histologic analyses, tumor tissues and major organs were fixed in 4% paraformaldehyde to prepare paraffin sections. Afterward, slices of tumor tissues were stained with hematoxylin and eosin (H&E) and TUNEL, and slices of major organs were also stained with H&E.

### 2.11 Immune response analyses in tumor

The harvested fresh tumor tissues were also subjected to immune cell and pro-inflammatory cytokine (IL-6, TNF-α, and IFN-γ) analyses. For immune cell analysis, tumor tissues were digested as single-cell suspension *via* enzymolysis by collagenase I/IV and filtration through a 40-μm mesh cell strainer (Jet Biofil, Guangzhou, China). After two washes with PBS containing 1% (w/v) bovine serum albumin (BSA), the resulting cells were stained with various fluorescence antibodies for FCM examinations. FITC-conjugated anti-CD11c (11-0114-81), PE-conjugated anti-CD86 (12-0862-81), and APC-conjugated anti-CD80 (17-0801-81) were used to label mature DC cells. PerCP-Cyanine5.5-conjugated anti-CD11b (45-0112-80) and FITC-conjugated anti-F4/80 (11-4801-82) were employed to stain macrophage cells. To evaluate T-cell subsets, PerCP-Cyanine5.5-conjugated anti-CD45 (45-0451-80), FITC-conjugated anti-CD3 (11-0032-82), PE-conjugated anti-CD8a (12-0081-82), and APC-conjugated anti-CD4 (17-0042-81) were leveraged. All of these fluorescence antibodies were purchased from eBioscience Inc. (San Diego, USA). The following FCM analysis was performed with an FCM instrument (BD FACS Calibur). When completing sample preparations by homogenization and centrifugation of tumor tissues, pro-inflammatory cytokines (IL-6, TNF-α, and IFN-γ) were quantified using commercial enzyme-linked immunosorbent assay (ELISA) kits (Quanzhou Ruixin Biotechnology Co., Ltd., China) as per the manufacturer’s instructions.

### 2.12 Hemolysis assay

Whole blood from a healthy C57BL/6 mouse was collected using an anticoagulant tube with citrate in it. Red blood cells (RBCs) were harvested from the bottom of the tube after centrifugation at 1,000×*g* for 5 min and then washed five times with PBS for hemolysis assay. RBC solution (200 μl) was mixed with different OMV solutions (200 μl, 1 mg/ml) to make sure the final concentration of OMVs was 0.5 mg/ml. Moreover, PBS and deionized (DI) water were also mixed with RBC solution for negative and positive control, respectively. After finishing the incubation at 37°C for 30 min with shaking at 300 rpm, the mixtures were centrifuged at 1,000×*g* for 5 min. Afterward, 200 μl of supernatant was transferred to a 96-well plate to measure the absorbance value at 570 nm using Spark^®^ Multimode Microplate Reader (TECAN, Switzerland). The hemolysis result was calculated using the following formula:


$$\begin{array}{c} \text{Hemolysis (\%) = (OMVs absorbance - PBS absorbance)/}\\ \;\;\;\text{(DI water absorbance - PBS absorbance) }\times 100 \end{array}$$

### 2.13 Statistical analysis

Results are presented as the mean ± standard deviation (SD). One-way analysis of variance (ANOVA) with Tukey’s *post-hoc* analysis was used to calculate the statistical significance among different groups. All statistical analyses were performed using GraphPad Prism 8. The significant difference was reflected by *p* < 0.05.

## 3 Results and discussion

### 3.1 Preparation and characterization of bioengineered OMVs

**Figure 1:**
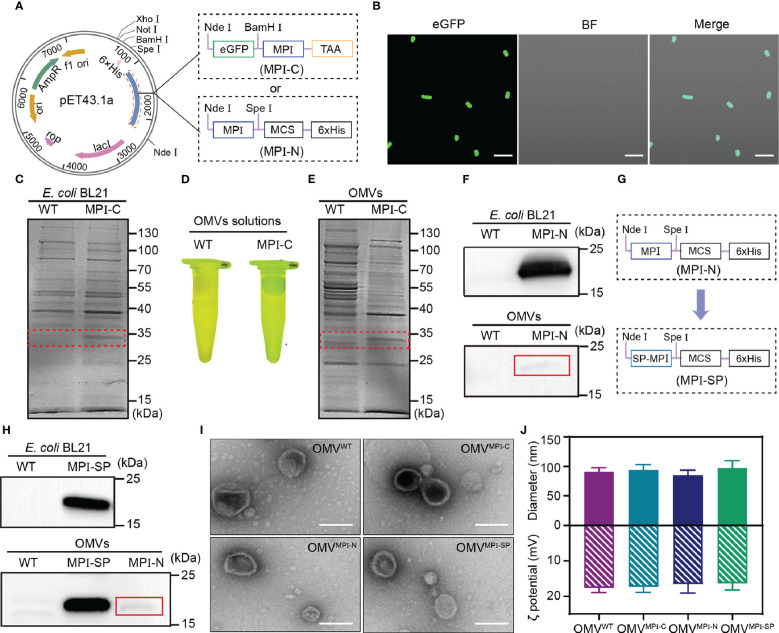
Preparation and characterization of OMVs. **(A)** Schematic representation showing constructions of pET43.1a-MPI-C and pET43.1a-MPI-N. **(B)** CLSM images of transformed *E. coli* (BL21) expressing eGFP-MPI (MPI-C) fusion peptide. BF, bright field. Scale bars = 10 μm. **(C)** SDS-PAGE analysis of total proteins from WT and transformed *E. coli* (BL21) expressing MPI-C fusion peptide. The red dashed box indicates the position of MPI-C. **(D)** Fluorescence image of OMV^WT^ and OMV^MPI-C^ solutions excited by blue light. **(E)** SDS-PAGE analysis of total proteins from OMV^WT^ and OMV^MPI-C^. The red dashed box indicates the position of MPI-C. **(F)** Western blot analysis of MPI-N expressions in transformed *E. coli* (BL21) and the corresponding OMVs. The red line box indicates the slight band of MPI-N. **(G)** Schematic representation of pET43.1a-MPI-SP construction. **(H)** Western blot analysis of MPI-SP expression in transformed *E. coli* (BL21) and the corresponding OMVs. MPI-N expression in OMVs shown in the bottom panel is used as a control. The red line box indicates the slight band of MPI-N. **(I)** TEM images of OMV^WT^, OMV^MPI-C^, OMV^MPI-N^, and OMV^MPI-SP^. Scale bars = 100 nm. **(J)** Histogram showing hydrodynamic sizes and zeta potentials of OMV^WT^, OMV^MPI-C^, OMV^MPI-N^, and OMV^MPI-SP^. Measurements with DLS were repeated three times. Data are presented as mean ± SD.

To prepare bioengineered OMVs containing the antitumor MPI fusion peptide, two recombinant plasmids, capable of expressing fusion peptides with MPI at the C (MPI-C) and N (MPI-N) terminal, respectively, as illustrated in **Figure 1A**, were constructed and then transformed into competent *E. coli* (BL21) cells. For MPI-C fusion peptide, a termination codon TAA was added immediately at the C terminal of MPI in order to prevent hexahistidine (6×His) tag from hindering the function of MPI. Due to the fused eGFP, expression of MPI-C in bioengineered bacteria was demonstrated *via* observing its fluorescence after they were induced by IPTG (**Figure 1B**). In addition, sodium dodecyl sulfate polyacrylamide gel electrophoresis (SDS-PAGE) analysis was also performed to identify the components of fusion peptide expressed in bacteria. Results exhibited in **Figure 1C** clearly indicated the existence of fusion peptide at the position of approximately 30 kDa in bioengineered bacteria compared with that in wild-type (WT) bacteria. Then, OMVs derived from WT and bioengineered bacteria were purified by serials of centrifugations and filtrations (see *Materials and Methods* for details). The fusion peptide MPI-C contained in bioengineered OMVs was confirmed with the same methods described above, and both of them gave satisfactory results (**Figures 1D, E**).

As illustrated in **Figure 1A**, MPI can fuse with the multiple clone site in pET43.1a to genetically express fusion peptide MPI-N with a 6×His tag at the C terminal that facilitates Western blot detection. Therefore, Western blot assay was applied to evaluate the MPI-N expression in bioengineered bacteria and the corresponding OMVs, respectively. It was very unexpected that they had abundant expressions in bacteria but slight expressions in OMVs (**Figures 1F**, **S1**). To further affirm this result, we also performed an SDS-PAGE analysis and obtained a similar conclusion (**Figure S2**). For the purpose of increasing the fusion peptide MPI-N in OMVs, a signal peptide (SP) derived from outer membrane protein A (OmpA) of *E. coli*, which is documented to facilitate the protein transfer from cytoplasm to OMVs (37, 38), was fused to the N terminal of MPI-N, and the new fusion peptide was renamed MPI-SP (**Figure 1G**). Ascribing from this SP, MPI-SP exhibited excellent expression performance both in bacteria and in OMVs verified by Western blot (**Figures 1H**, **S3**) and SDS-PAGE analysis (**Figure S4**). It is noteworthy that MPI-SP had an absolutely higher expression than MPI-N in OMVs (**Figure 1H**, bottom panel, **Figure S3**).

The above results manifested that we successfully fabricated three kinds of engineered OMVs: encapsulated MPI-C, MPI-N (slight amount), and MPI-SP, respectively. To characterize their morphological features, imaging by TEM was performed and all of them emerged as spherical nanoscale vesicles (**Figure 1I**). Dynamic light scattering (DLS) results reported that they had uniform diameters of approximately 90 nm and zeta potentials of approximately −17 mV (**Figure 1J**). These indicators demonstrated that bioengineered OMVs had no alteration on morphology after incorporation of a heterologous protein.

### 3.2 Bioengineered OMVs inhibit BCa progress *in vitro*

**Figure 2:**
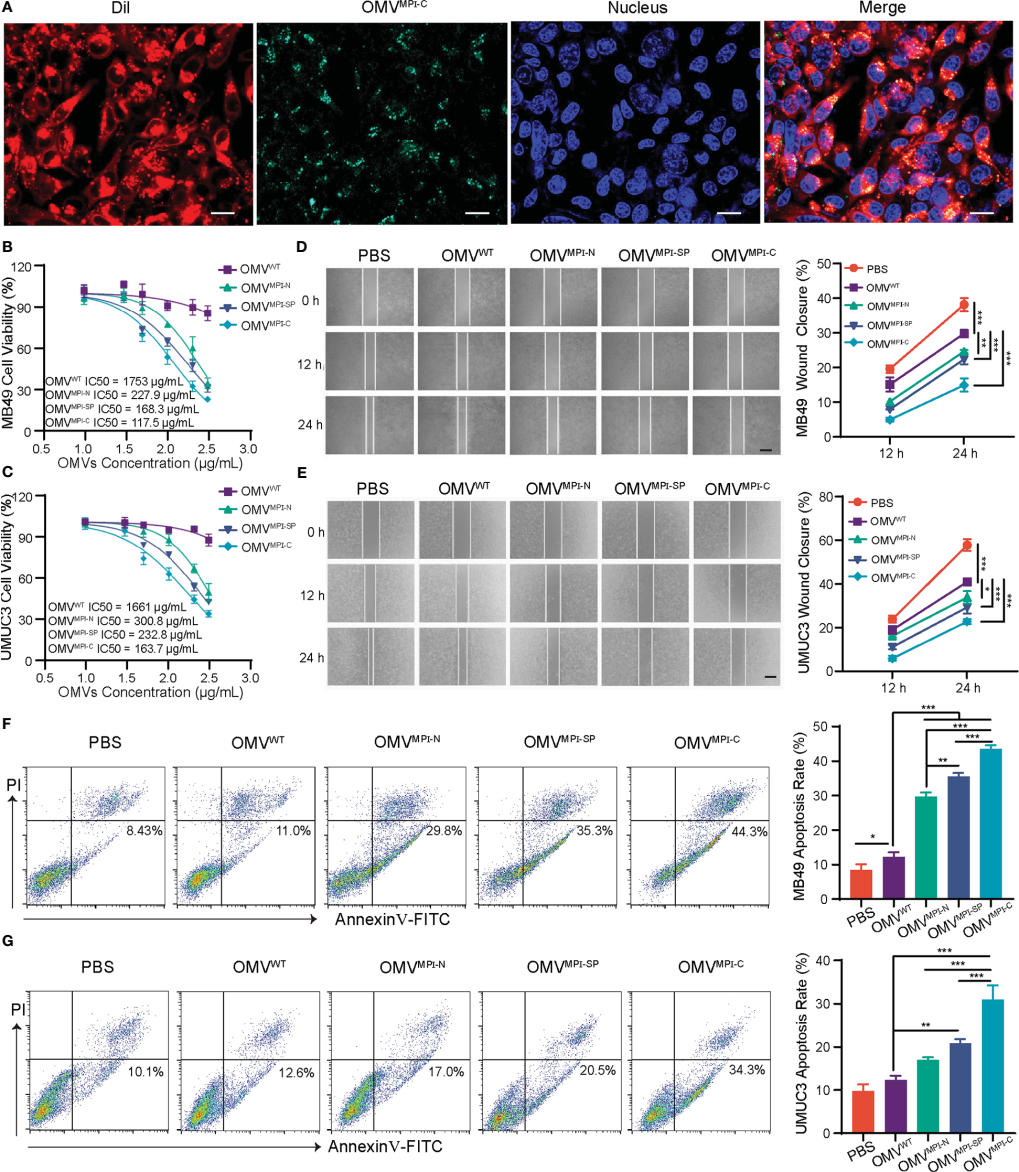
The antitumor effects of bioengineered OMVs *in vitro*. **(A)** CLSM images representing DiL-labeled OMV^MPI-C^ endocytosis by MB49 cells after 6-h incubation. Cell nuclei were stained with Hoechst 33342 (blue). Scale bar = 20 μm. IC_50_ curves of MB49 **(B)** and UMUC3 **(C)** after treatment with different OMV formulations for 24 h (*n* = 3). Representative images and quantification analysis of MB49 **(D)** and UMUC3 **(E)** cell wound-healing assay after treatment with different OMV formulations (100 μg/ml). Images were obtained at 0, 12, and 24 h (*n* = 3). Scale bars = 100 μm. Representative FCM results and corresponding quantification analysis showing the percentages of apoptotic MB49 **(F)** and UMUC3 **(G)** cells after treatment with different OMV formulations (100 μg/ml) for 48 h (*n* = 3). Data in **(B–G)** are presented as mean ± SD. ns, no significance; *p < 0.05, **p < 0.01, ***p < 0.001.

To investigate the effectiveness of bioengineered OMVs upon anticancer *in vitro*, two BCa cells with different sources including a murine MB49 and a human UMUC3 were employed due to the selective antiproliferative effect of MPI against various BCa cell lines (9). Initially, uptake of OMVs by tumor cells was verified using a CLSM by incubating DiL-labeled OMV^MPI-C^, which has green fluorescence, and MB49 cells for 6 h. **Figure 2A** clearly exhibited the co-localization of green and red fluorescence in tumor cells, indicating the endocytosis of OMVs. Afterward, cell viability assays of MB49 and UMUC3 cells were performed to evaluate the tumor inhibition ability of bioengineered OMVs. Both of them had a pronounced reduction of their viabilities when treated with bioengineered OMVs (OMV^MPI-N^, OMV^MPI-SP^, and OMV^MPI-C^) for 24 h when compared with OMV^WT^ treatment, while OMV^MPI-C^ had the lowest half-maximal inhibitory concentration (IC_50_) (**Figures 2B, C**). However, a non-carcinomatous cell named bEnd.3, derived from mouse brain microvascular endothelial cells, exhibited no viability suppression when being treated with different OMVs at the concentration of <100 μg/ml (**Figure S5**), indicating the specific destruction ability of MPI fusion peptides in bioengineered OMVs to tumor cells. Similarly, the tumor migration performance of MB49 and UMUC3 cells resulting from wound-healing assays was restrained due to the incubation of OMV^MPI-N^, OMV^MPI-SP^, or OMV^MPI-C^, and the effectiveness was more apparent when the duration prolonged to 24 h (**Figures 2D, E**). In addition, the apoptotic tumor cells caused by the treatment of bioengineered OMVs were analyzed by FCM. The results presented in **Figure 2F** manifested the percentage of apoptotic MB49 cells increased to 44.3% in OMV^MPI-C^ group, much higher than the 8.43% in PBS group and 11.0% in OMV^WT^ group, respectively. With regard to UMUC3 cells, we unsurprisingly obtained a very similar result (**Figure 2G**). To further verify that the tumor cytotoxicity was derived from the expression of MPI in OMVs, we incubated OMVs only containing eGFP (OMV^eGFP^), the fragment of MPI-C fusion protein after the removal of MPI, as a negative control with both tumor cells, respectively. The results shown in **Figure S6** manifested the loss of tumor cytotoxicity of OMV^eGFP^. In addition, tumor apoptosis detection by FCM could draw the same conclusion (**Figures S7**, **S8**). In accordance with the above results, we can conclude that the bioengineered OMVs fabricated here are capable of efficiently limiting BCa progress *in vitro*. Although all the three bioengineered OMVs are endowed with some anticancer properties *via* genetically expressing MPI-C, MPI-N, or MPI-SP in them concluded from the above results, OMV^MPI-C^ always has a superior performance and OMV^MPI-N^ is the weakest comparatively. Apart from the encapsulated abundance such as only a slight amount of MPI-N in OMV^MPI-N^, this difference is also speculated to be ascribed to the construction of fusion peptides. MPI is located at the C terminal of MPI-C without any obstruction, facilitating MPI to interact with cancer cell membrane. However, SP and eGFP locating at the N and C terminal of MPI-SP respectively may hinder its interaction with cancer cell membrane.

### 3.3 *In vivo* investigation of bioengineered OMVs for BCa treatment

**Figure 3:**
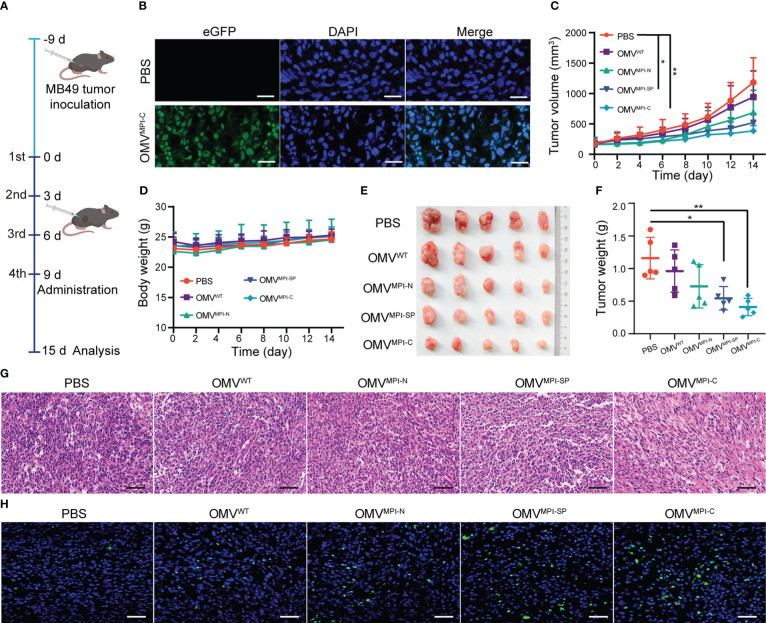
Investigation of bioengineered OMVs for BCa treatment *in vivo*. **(A)** The diagrammatic sketch of experimental design for evaluating BCa inhibition effectiveness of bioengineered OMVs using subcutaneous tumor-bearing mice. **(B)** Fluorescence images of tumor slice after intratumoral injection of PBS and OMV^MPI-C^ solutions. Cell nuclei were stained with DAPI (blue). Scale bar = 50 μm. Subcutaneous tumor volumes **(C)** and body weights **(D)** of tumor-bearing mice recorded every other day from day 0 to day 15 (*n* = 5). Image **(E)** and corresponding weights **(F)** of tumors harvested from tumor-bearing mice at the end of the treatments (*n* = 5). H&E staining **(G)** and fluorescence TUNEL staining images **(H)** of tumor tissue at the end of the treatments. Scale bars = 50 μm. Data in **(C, D, F)** are presented as mean ± SD. Statistical significance was calculated *via* one-way ANOVA with a Tukey’s *post-hoc* test. *p < 0.05, **p < 0.01.

Given the good performance of bioengineered OMVs upon BCa inhibition *in vitro*, we subsequently assessed their effectiveness to cure BCa *in vivo* using MB49 tumor-transplanted mice models. For this purpose, MB49 cells were subcutaneously inoculated into C57BL/6 mice with 1 × 10^6^ cells per mouse, and tumors were allowed to grow until volumes reached approximately 100 mm^3^ before OMV treatments. The tumor-bearing mice were divided into five groups (five mice in each group) and different intratumor injection regimes of PBS, OMV^WT^, OMV^MPI-N^, OMV^MPI-SP^, and OMV^MPI-C^ were repeated four times with a frequency of 3 days/time as illustrated in **Figure 3A**. To confirm that the injected OMVs can be phagocytized by tumor cells, we observed green fluorescence of the representative OMV^MPI-C^ in the tumor histological section after 12-h administration, which are shown in **Figure 3B**. Tumor growth curves shown in **Figure 3C** depicted different therapy efficacies of OMVs in the monitoring period. Although OMV^WT^ and OMV^MPI-N^ presented slight inhibition potency, OMV^MPI-SP^ and OMV^MPI-C^ treatments resulted in better therapeutic outcomes with statistical significance when compared with the PBS group, suggesting the anticancer activity of the MPI fusion peptide in bioengineered OMVs. In the meantime, almost no body weight loss of tumor-bearing mice in treatment groups was observed (**Figure 3D**). Both the images of tumors resected at the endpoint of administration and the corresponding tumor weight showed the optimum tumor inhibition efficacy of OMV^MPI-C^ (**Figures 3E, F**), which was consistent with the measurement *in vivo*. Furthermore, the excised tumor tissues were subjected to histological analysis. From the images of H&E-stained sections, we observed only small areas of necrosis after treatment with OMV^WT^, OMV^MPI-N^, or OMV^MPI-SP^, whereas OMV^MPI-C^ elicited larger necrosis areas of tumor tissues (**Figure 3G**). Immunofluorescence of TUNEL further revealed the strongest tumoricidal effect of the OMV^MPI-C^ formulation (**Figure 3H**). Together, these results indicate that the facilely fabricated OMV^MPI-C^ possesses a great potential in BCa therapy.

### 3.4 Immune cell infiltration induced by OMVs

**Figure 4:**
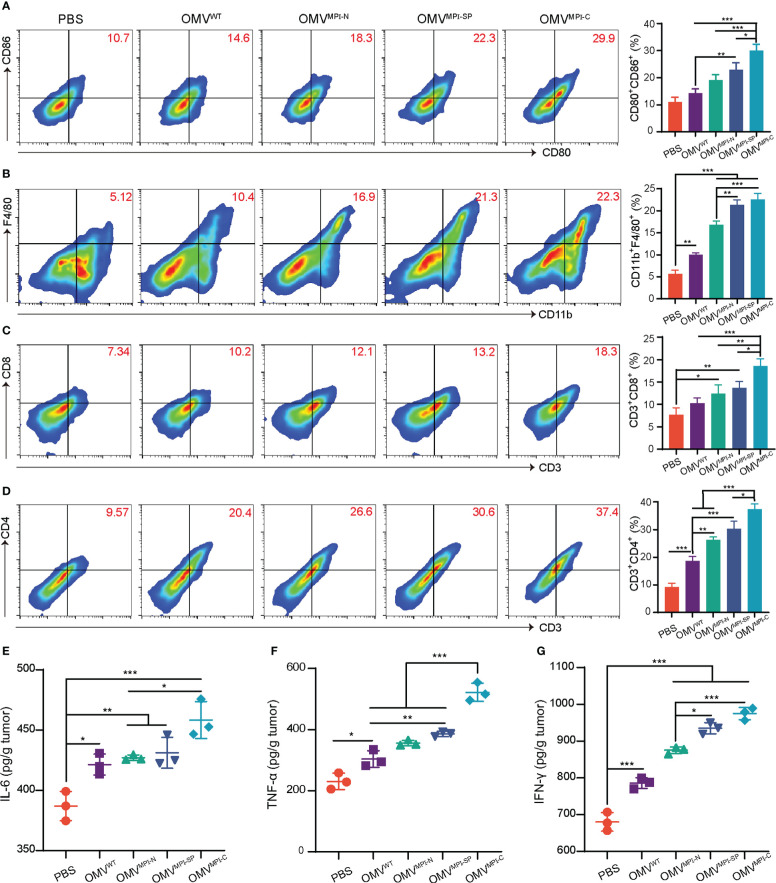
Stimulation of antitumor immunity by bioengineered OMVs. **(A)** Representative FCM analysis results of mature DCs (CD80^+^CD86^+^ gated on CD11c^+^) and the corresponding percentages of CD80^+^CD86^+^ DCs in tumors (*n* = 3). **(B)** Representative FCM analysis images of CD11b^+^F4/80^+^ macrophages and the corresponding percentages in MB49 tumors (*n* = 3). Representative FCM plots of CD3^+^CD8^+^ **(C)** and CD3^+^CD4^+^ **(D)** T lymphocyte infiltration (both gated on CD45^+^) and the corresponding quantitative analysis (*n* = 3). The levels of pro-inflammatory cytokines including IL-6 **(E)**, TNF-α **(F)**, and IFN-γ **(G)** in tumor tissues after treatment with different OMV formulations (*n* = 3). Data are presented as mean ± SD. Statistical significance was calculated *via* one-way ANOVA with a Tukey’s *post-hoc* test. *p < 0.05, **p < 0.01, ***p < 0.001.

Naturally derived OMVs have been well documented as immunostimulation reagents applied in cancer treatment due to various pathogen-associated molecular patterns, such as lipopolysaccharide and lipoprotein (20). Therefore, it is speculated that the excellent anticancer performance of bioengineered OMVs not only results from the encapsulated fusion peptide MPI, but also ascribes from immune cell infiltration in the tumor microenvironment induced by OMVs. To understand the alteration of immune cell infiltration in the tumor microenvironment after receiving administrations of different OMV formulations, we examined the specific markers of several immune cells and the associated pro-inflammatory cytokines. As illustrated in **Figure 4A**, the surface expression markers of CD86^+^ and CD80^+^ on CD11c^+^ DCs had an observable rise in OMV treatment groups, indicating the transformation of immature DCs into mature DCs. Moreover, macrophages (CD11b^+^ and F4/80^+^) were also recruited into the tumor microenvironment because of the OMV management (**Figure 4B**). Infiltration of T lymphocytes is another important indicator for evaluating immunostimulation in tumor. According to **Figures 4C, D**, the therapeutic outcome from OMVs exhibited an enhanced CD8^+^ and CD4^+^ T-cell infiltration in tumor tissues. The representative gating strategies of the above FCM plots are shown in **Figures S9**–**S12**, respectively. The above FCM results revealed that OMVs were capable of inducing various immune cell infiltrations in the tumor microenvironment. In addition, we found that OMV^MPI-C^ always has a better performance than OMV^MPI-N^ or OMV^MPI-SP^ with respect to immune cell infiltration, which was consistent with the therapeutic outcomes *in vitro* and *in vivo*. Given that pro-inflammatory cytokines such as IL-6, TNF-α, and IFN-γ enable the initiation of immune responses and the inhibition of tumor growth, we subsequently evaluated the yields of these cytokines in tumor tissues. Similar to the detection results of immune cells, the level of these three pro-inflammatory cytokines pronouncedly increased in bioengineered OMV groups compared to control groups including PBS and OMV^WT^ (**Figures 4E–G**). Collectively, these results manifested that bioengineered OMVs, especially OMV^MPI-C^, are competent for ameliorating the tumor microenvironment by inducing immune cell infiltration and the release of pro-inflammatory cytokines, leading to the inhibition of tumor growth.

### 3.5 Biosafety evaluation of bioengineered OMVs

**Figure 5:**
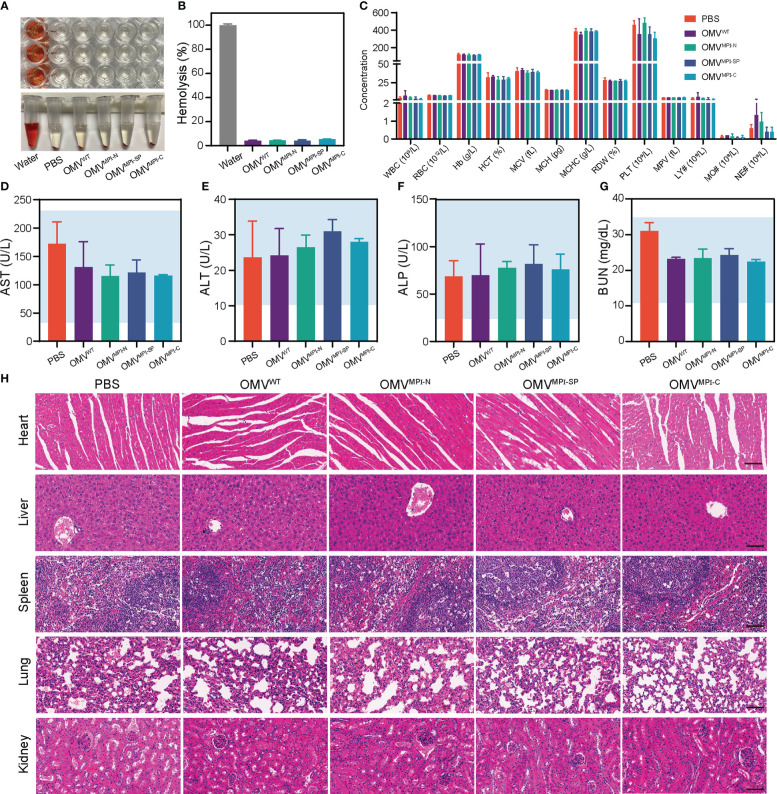
Biosafety evaluation of bioengineered OMVs. **(A)** Hemolytic images of OMV^WT^, OMV^MPI-N^, OMV^MPI-SP^, and OMV^MPI-C^ in tubes and plates. **(B)** The calculated hemolysis percentages of bioengineered OMVs (*n* = 3). **(C)** Blood routine examination results of tumor-bearing mice from different treatment groups (*n* = 3). **(D–G)** Serum biochemical indexes of AST, ALT, ALP, and BUN of tumor-bearing mice from different treatment groups (*n* = 3). Ranges indicated by blue are normal ranges and all the detected serum biochemical indexes were in normal ranges. **(H)** Histology H&E staining analysis of heart, liver, spleen, lung, and kidney slices obtained from tumor-bearing mice after different treatments. Scale bars = 50 μm. Data in **(B–G)** are presented as mean ± SD.

Although application of bacteria for tumor therapy has attracted widespread interest over the past few decades due to the development of cancer immunology, the potential toxicity severely limits their extensive application (39). Their uncontrollable proliferation in an organism after bacteria treatment may cause hemolysis and high fever, even mortality (40). In contrast, OMVs naturally secreted by bacteria display satisfactory therapeutic efficacy with a comparatively higher biocompatibility in the premise of the appropriate dosage (20). Herein, in order to assess the biocompatibility of bioengineered OMVs, multiple indicators of tumor-bearing mice were examined at the end of OMV administration. The hemolysis assay was first performed with the RBCs isolated from blood of normal C57BL/6 mice. After incubation, with deionized water serving as the positive control group and PBS solution as the negative control group, the calculated hemolysis ratio of OMV groups was lower than 5% (**Figures 5A, B**), indicating the negligible hemolysis caused by bioengineered OMVs *in vitro*. Subsequently, blood routine examination and biochemical indicator quantification were conducted for the evaluation of systemic toxicity. Results of blood routine examination revealed that all blood indicators had no significant alteration in OMV treatment groups when compared with the PBS group (**Figure 5C**). The levels of serum biochemical indicators of liver and kidney function (including AST, ALT, ALP, and BUN) were located at normal range (**Figures 5D–G**). H&E-stained sections of major organs including heart, liver, spleen, lung, and kidney resected from tumor-bearing mice after the experiments demonstrated no visible lesion caused by the repeated administration of bioengineered OMVs (**Figure 5H**). All of these lines of evidence suggested that our fabricated OMVs showed great biocompatibility and ignorable systemic toxicity, facilitating future biomedical applications.

## 4 Conclusion

In the present study, we have successfully fabricated a versatile antitumor formulation with high performance by leveraging bacterial OMVs as nano-carriers to encapsulate an MPI fusion peptide generated by genetic engineering technology. In consideration of the tumoricidal mechanism of MPI, we fused MPI at the C and N terminal of the fusion peptide, respectively, facilitating MPI to embed into the cell membrane, whereas an SP from OmpA has to be introduced because of the unimplemented encapsulation of MPI-N into OMVs. Thus, we obtained three kinds of bioengineered OMVs ultimately, namely, OMV^MPI-N^ (only slight MPI-N in OMVs), OMV^MPI-SP^ (MPI-N blocked by SP), and OMV^MPI-C^. Among them, it was predictable that OMV^MPI-C^ always exhibited optimal efficiency against BCa in both *in vitro* and *in vivo* experiments, expanding the application of MPI to cancer chemotherapy. Such fusion expression of peptide can be readily generalized to other functional peptides, offering a facile and low-cost strategy for loading therapeutic peptides into OMVs and further delivery into target cells. In the aspect of cancer treatment, combining the chemotherapy of MPI with the intrinsic immunostimulation of OMVs, capable of converting cold tumor to hot tumor, further improved the therapeutic outcomes in BCa mouse models. Lastly, we demonstrated the fantastic biocompatibility of bioengineered OMVs in mice *via* management of intratumor injection, while a severe immune storm may be triggered by intravenous injection reported by a previous study (20). In other words, nanosized OMVs with an enhanced permeability and retention effect are absolutely biosafe through the method of local administration, suggesting that they have great potential to become a novel reagent for intravesical instillation after transurethral surgical intervention of BCa.

## Data availability statement

The original contributions presented in the study are included in the article/**Supplementary Material**, further inquiries can be directed to the corresponding authors.

## Ethics statement

The animal study was reviewed and approved by Animal Use and Care Administrative Advisory Committee of Shenzhen Luohu Hospital Group.

## Author contributions

CR, YL, and ZC conceived the idea and designed the research. All experiments were conducted by CR, and ZL and LX helped analyze the data. CR wrote the manuscript. YL and SW reviewed and revised the manuscript. All authors contributed to the article and approved the submitted version.
