# Supplementary material for: Bioengineered bacterial outer membrane vesicles encapsulated Polybia–mastoparan I fusion peptide as a promising nanoplatform for bladder cancer immune-modulatory chemotherapy
Source: Front Immunol. 2023 Mar 14;14:1129771. doi: 10.3389/fimmu.2023.1129771 (PMC10043419; doi:10.3389/fimmu.2023.1129771)
Supplement: Supplementary file 1 [file DataSheet_1.docx]

Supplementary Material for

Bioengineered bacterial outer membrane vesicles encapsulated Polybia-mastoparan I fusion peptide as a promising nanoplatform for bladder cancer immune-modulatory chemotherapy

Chunyu Ren et al.

## Supplementary Figures


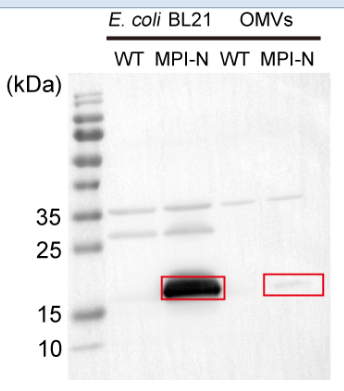


**Supplementary Figure 1.** Western blot analysis of MPI-N expression in transformed *E. coli* (BL21) and the corresponding OMVs. Red line box indicates the position of MPI-N. (This is the original image of Figure 1 F.)


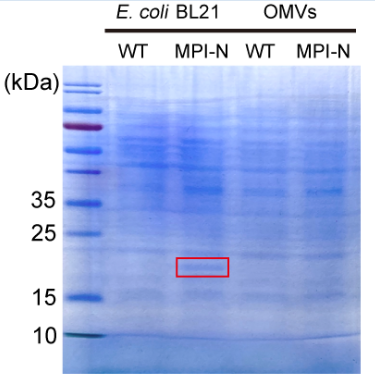


**Supplementary Figure 2.** SDS-PAGE analysis of total proteins from MPI-N expression in transformed *E. coli* (BL21) and the corresponding OMVs. Red line box indicates the position of MPI-N.


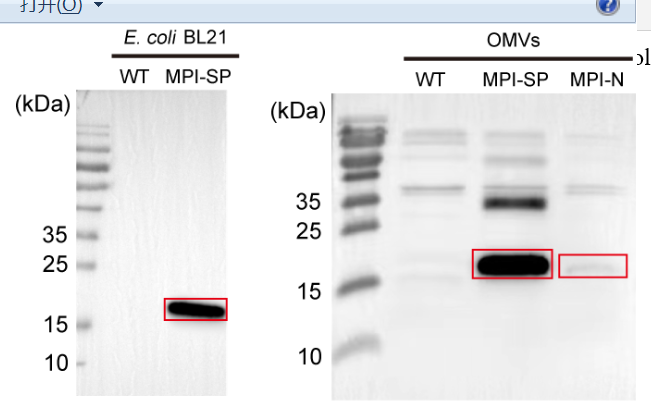


**Supplementary Figure 3.** Western blot analysis of MPI-SP expression in transformed *E. coli* (BL21) and MPI-SP and MPI-N expression in OMVs. Red line box indicates the position of MPI-SP or MPI-N. (This is the original image of Figure 1 H.)


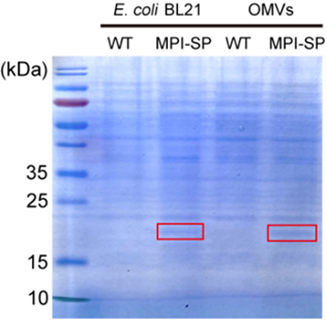


**Supplementary Figure 4.** SDS-PAGE analysis of total proteins from MPI-SP expression in transformed *E. coli* (BL21) and the corresponding OMVs. Red line box indicates the position of MPI-SP.


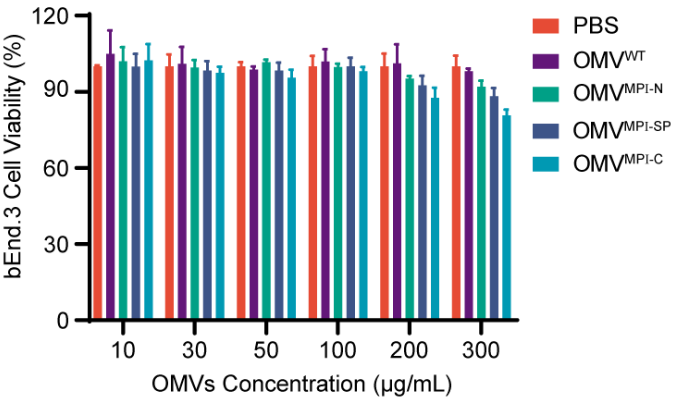


**Supplementary Figure 5.** Cell viability of bEnd.3 after treatment with different OMVs concentrations for 24 h (n = 3).


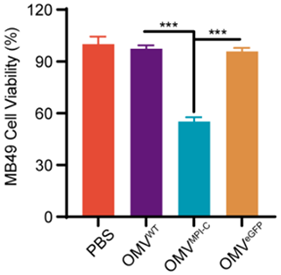

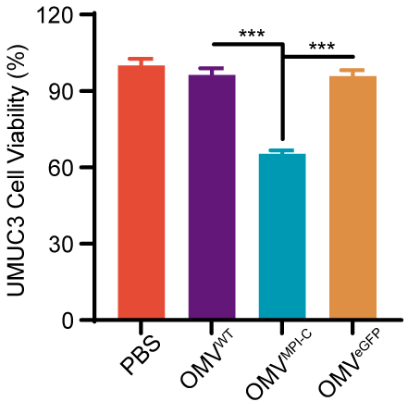


**Supplementary Figure 6.** Cell viabilities of MB49 (left), and UMUC3 (right) after treatment with different OMVs formulations (100 μg/mL) for 24 hours (n = 3).


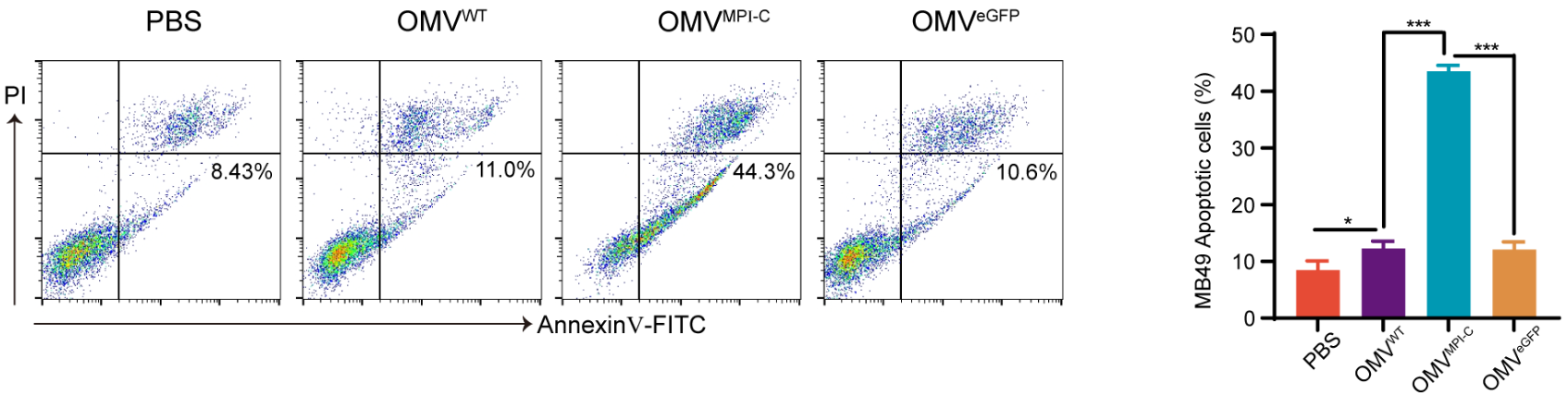
 **
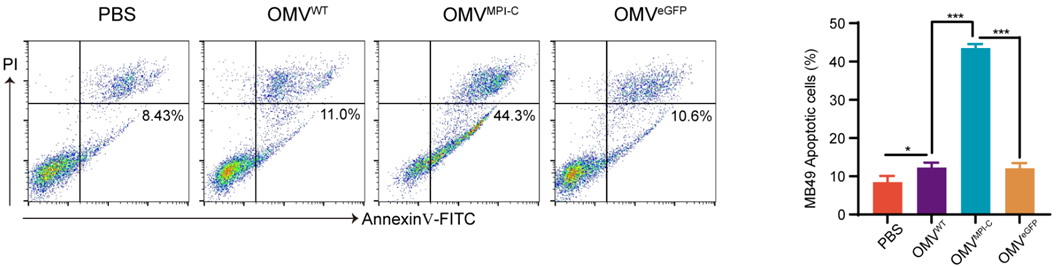
**

**Supplementary Figure 7.** Representative FCM results and corresponding quantification analysis showing the percentages of apoptotic MB49 cells after treatment with different OMVs formulations (100 μg/mL) for 48 hours (n = 3).


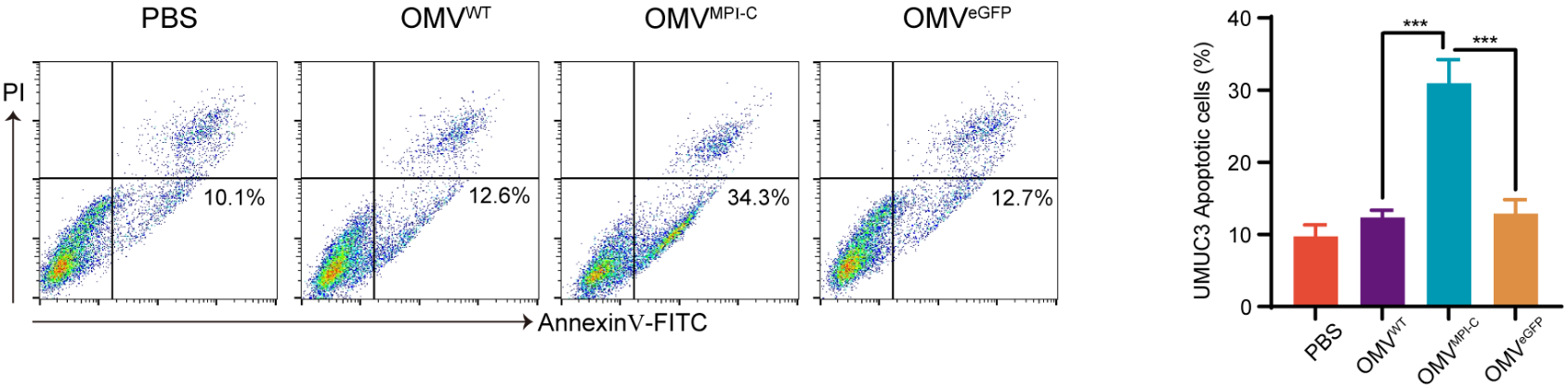
 **
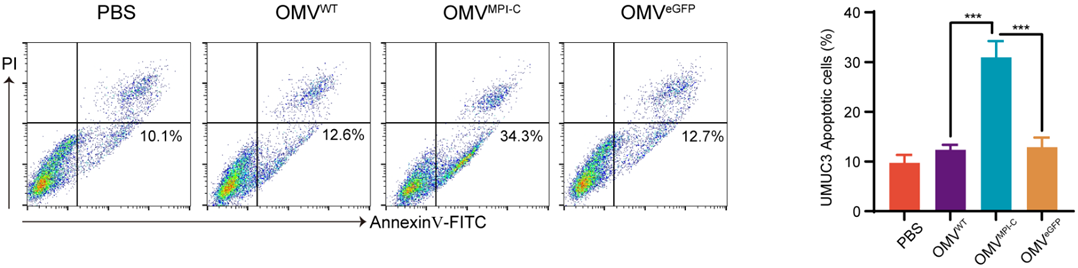
**

**Supplementary Figure 8.** Representative FCM results and corresponding quantification analysis showing the percentages of apoptotic UMUC3 cells after treatment with different OMVs formulations (100 μg/mL) for 48 hours (n = 3).


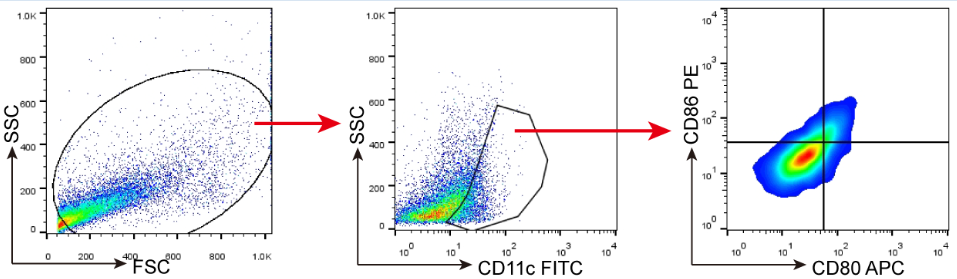


**Supplementary Figure 9.** Representative flow cytometry gating strategies for the experiments in Figure 4 A (n = 3).


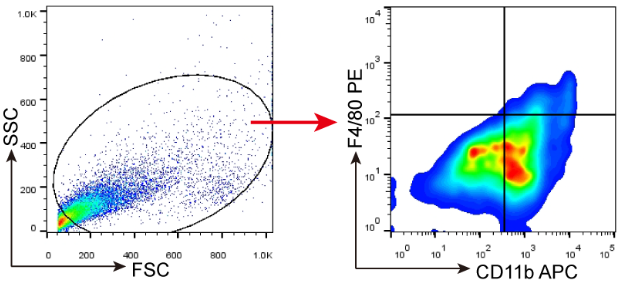


**Supplementary Figure 10.** Representative flow cytometry gating strategies for the experiments in Figure 4 B (n = 3).


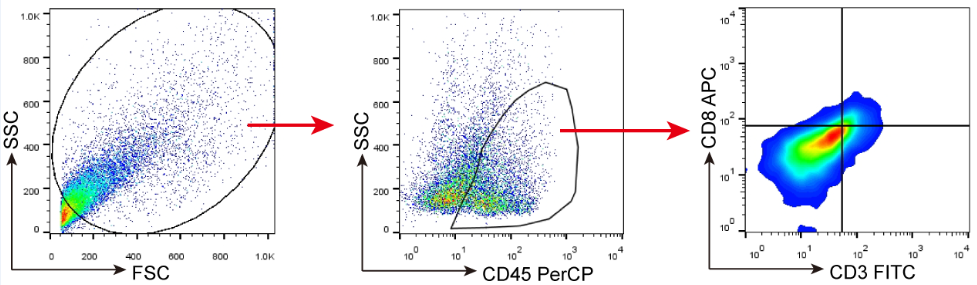


**Supplementary Figure 11.** Representative flow cytometry gating strategies for the experiments in Figure 4 C (n = 3).


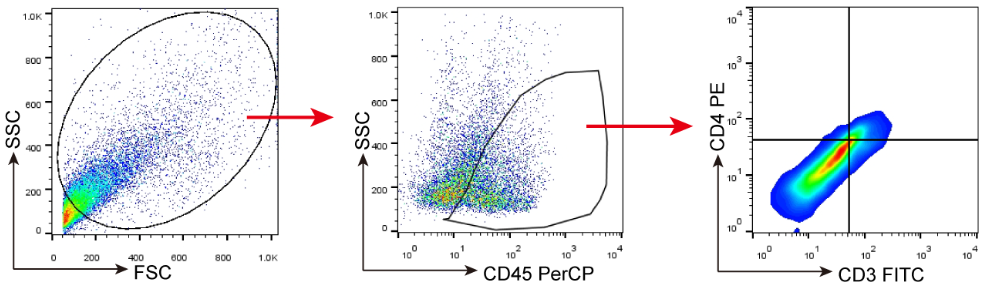


**Supplementary Figure 12.** Representative flow cytometry gating strategies for the experiments in Figure 4 D (n = 3).
